# The preventive services use self-efficacy (PRESS) scale in older women: development and psychometric properties

**DOI:** 10.1186/s12913-016-1321-x

**Published:** 2016-02-20

**Authors:** Mini E. Jacob, Wei-Hsuan Lo-Ciganic, Laurey R. Simkin-Silverman, Steven M. Albert, Anne B. Newman, Lauren Terhorst, Joni Vander Bilt, Janice C. Zgibor, Elizabeth A. Schlenk

**Affiliations:** Graduate School of Public Health Department of Epidemiology, University of Pittsburgh, Pittsburgh, USA; College of Pharmacy Department of Pharmacy, University of Arizona, Tucson, USA; Graduate School of Public Health Department of Behavioral and Community Health Sciences, University of Pittsburgh, Pittsburgh, USA; School of Health and Rehabilitation Sciences Department of Occupational Therapy, University of Pittsburgh, Pittsburgh, USA; School of Nursing Department of Health and Community Systems, University of Pittsburgh, Pittsburgh, USA

**Keywords:** Self-efficacy, Preventive services, Older adults, Psychometrics

## Abstract

**Background:**

Preventive services offered to older Americans are currently under-utilized despite considerable evidence regarding their health and economic benefits. Individuals with low self-efficacy in accessing these services need to be identified and provided self-efficacy enhancing interventions. Scales measuring self-efficacy in the management of chronic diseases exist, but do not cover the broad spectrum of preventive services and behaviors that can improve the health of older adults, particularly older women who are vulnerable to poorer health and lesser utilization of preventive services. This study aimed to evaluate the psychometric properties of a new preventive services use self-efficacy scale, by measuring its internal consistency reliability, assessing internal construct validity by exploring factor structure, and examining differences in self-efficacy scores according to participant characteristics.

**Methods:**

The Preventive Services Use Self-Efficacy (PRESS) Scale was developed by an expert panel at the University of Pittsburgh Center for Aging and Population Health - Prevention Research Center. It was administered to 242 women participating in an ongoing trial and the data were analyzed to assess its psychometric properties. An exploratory factor analysis with a principal axis factoring approach and orthogonal varimax rotation was used to explore the underlying structure of the items in the scale. The internal consistency of the subscales was assessed using Cronbach’s alpha coefficient.

**Results:**

The exploratory factor analysis defined five self-efficacy factors (self-efficacy for exercise, communication with physicians, self-management of chronic disease, obtaining screening tests, and getting vaccinations regularly) formed by 16 items from the scale. The internal consistency of the subscales ranged from .81 to .94. Participants who accessed a preventive service had higher self-efficacy scores in the corresponding sub-scale than those who did not.

**Conclusions:**

The 16-item PRESS scale demonstrates preliminary validity and reliability in measuring self-efficacy in the use of preventive services among older women. It can potentially be used to evaluate the impact of interventions designed to improve self-efficacy in the use of preventive services in community-dwelling older women.

## Background

Despite clear guidelines set by the U.S. Preventive Services Task Force and recent legislative and policy measures intended to improve access to health care, there are significant gaps in the utilization of preventive services by older Americans [1]. Improving access to preventive services like routine disease screening and scheduled vaccinations can have substantial benefits. A uniform increase in the utilization of 9 clinical preventive services (control of hypertension, control of elevated LDL cholesterol, aspirin chemoprophylaxis, smoking cessation, colonoscopy screening, mammography screening, pap smear screening, influenza vaccination, and pneumococcal vaccination) to levels achieved by high performing health systems could prevent 50,000 to 100,000 deaths every year in the population aged less than 80 years [2]. Increasing the use of these services from current levels to 90 % would also result in total savings of $3.7 billion [3]. Closing this gap for older populations will require concerted action by the forces of public health infrastructure, community-based organizations, and aging services network. Efforts should particularly target older women who comprise 56 % of this population, and are vulnerable to poorer health and lesser utilization of preventive services [4].

Strategies to improve preventive service use should include public health interventions that extend beyond clinical settings and reach people individually or collectively to enhance community capacity [5, 6]. Such preventive health interventions are likely to be most effective if reliable and valid tools can be used to identify individuals and communities at risk for inadequate use of preventive services. An efficient method of risk-identification would include the assessment of self-efficacy in this domain. Self-efficacy, defined as individuals’ assessments of their ability to perform a specific behavior successfully [7], is an important belief that can predict health behaviors [8–11]. All major theories of health behavior change incorporate self-efficacy as a major component [12, 13]. Over the years, there has been a growing acceptance of the role of self-efficacy in modulating health behaviors and in turn positively affecting health outcomes. Research has shown that individuals with higher self-efficacy are more effective in the self-management of diabetes [14], hypertension [15], and arthritis pain [16]. High self-efficacy is also associated with better oral health [17] and better self-reported health in cardiovascular disease [18].

Self-efficacy plays an important role in determining the utilization of preventive services. It has been shown to be a strong predictive factor in a woman’s decision to obtain a mammogram [19] and to be associated with the probability of obtaining a colonoscopy for colon cancer screening [20]. Interventions that improve self-efficacy have also been shown to improve preventive health behaviors, such as physical activity [21]. Besides self-efficacy, there are multiple factors that determine the use of preventive services. While health insurance and economic access may be important factors in countries like Mexico [22], in the US, where preventive services for older adults are covered by Medicare, determinants of preventive service use among older adults include gender [4], race/ethnicity [23], depressive symptoms [24], health literacy, geographic isolation [25], as well as provider recommendation [26]. Greater aging satisfaction has been shown to be associated with greater use of preventive services [27]. Further investigation is required for understanding how the different predictors including self-efficacy may interplay in the final decision to access preventive care or participate in a preventive behavior; theoretical models include self-efficacy as an intermediate factor in the relationship between health literacy and health behavior [28]. However, the paramount role of self-efficacy in undeniable; enhancing self-efficacy is crucial for behavior change [7]. Yet, despite the considerable interest in lifestyle change for preventing death and disease in the recent years, the construct of self-efficacy in a broad range of preventive behaviors has not been given due importance by health researchers and health care personnel.

Several instruments have been developed to measure general self-efficacy as well as specific self-efficacy in domains as varied as breast feeding [29], exercise [30], smoking cessation [31], peer-aggression coping, and pain [32]. Self-efficacy in different domains of functioning may not correlate; therefore, it is important to develop self-efficacy measurement tools in specific domains [33]. Scales measuring self-efficacy in the management of chronic diseases exist [34], but do not cover the broad spectrum of preventive services and preventive behaviors that can improve the health of older adults in general and older women in particular. For example, the Stanford Chronic Disease Self-efficacy Scale assesses an individual’s confidence in self-managing disease symptoms, pain, and emotional distress caused by disease, but does not include items pertaining to self-efficacy for smoking cessation, cancer screening or obtaining immunizations. An instrument measuring self-efficacy in a wide range of preventive behaviors would be very useful in identifying high-risk individuals and may also help evaluate the effect of interventions designed to improve self-efficacy in this domain.

The University of Pittsburgh Center for Aging and Population Health - Prevention Research Center (CAPH-PRC) developed the Preventive Service Use Self-Efficacy (PRESS) Scale based on important areas of disease prevention for this age group known as the “10 Keys”™ to Healthy Aging (“10 Keys”^TM^). The “10 Keys”™, identified by experts in aging research at the CAPH-PRC, using evidence from epidemiological, clinical and lab studies, includes the following - control of (1) blood pressure, (2) glucose, and (3) low-density lipoprotein cholesterol (LDL-C), (4) smoking cessation, (5) cancer screenings, (6) immunizations, (7) physical activity, (8) musculoskeletal health, (9) social contact, and (10) combating depression. Seven of these keys have been listed by the National Commission on Prevention Priorities among the top 20 evidence-based clinical preventive services with the biggest impact on the health of the U.S. population [3]. A behavioral activation program based on the “10 Keys”™ has been found to be effective in improving diverse indicators of preventive health [35, 36]. The need for promotion and further evaluation of this evidence-based and effective program as an intervention to improve self-efficacy in preventive services use among older adults in the community led to the development of the PRESS scale.

For developing the scale, an expert panel consisting of 5 research faculty at the CAPH-PRC identified 21 questions pertaining to self-efficacy in preventive service use and preventive behaviors important for older adults. The initial item pool was generated based on a review of the literature and review of scales available in this domain. The expert panel developed 13 items to address each aspect of the “10 Keys”™. The remaining eight items were adapted from the Stanford Patient Education Research Center Chronic Disease Self-efficacy scale and assessed self-efficacy for exercising regularly, getting information about disease and disability prevention from community resources, and communicating with physicians [37]. A Likert scale response format was employed with graded scores ranging from 1 to 10, with 1 indicating “not at all confident” and 10 indicating “totally confident”. The 10 point scale was chosen considering the educational level of the participants and the success of other 10 point scales used in other studies conducted at the CAPH-PRC. Participants with higher scale scores are defined as having higher self-efficacy, that is, they have greater confidence in their ability to perform the indicated preventive behaviors.

In this study, we aimed to assess the internal construct validity of this scale by exploring its factor structure and examine differences in self-efficacy scores according to participant characteristics. We hypothesized that participants who reported participation in a certain preventive behavior would have higher self-efficacy scores for items pertaining to that behavior whereas participants who had a certain chronic disease would have lower self-efficacy scores for corresponding items compared to those who did not have the disease. We also aimed to assess the internal consistency reliability of this new scale. Our investigation was performed to show that the new scale can be used in community settings to identify women in need of strategies to enhance self-efficacy for preventive service use and adopt preventive behaviors.

## Methods

### Setting and sample

We conducted secondary analysis of baseline data from 242 women participating in a prevention trial integrating the Arthritis Foundation Exercise Program (AFEP) with the “10 Keys”™ program, a result of the collaboration between the Arthritis Foundation of Pennsylvania and the CAPH-PRC. This cluster randomized controlled trial is evaluating the effectiveness of the integrated program (AFEP + “10 Keys”™) in improving arthritis symptoms, self-efficacy, indicators of preventive services use, and risk factors for disability and chronic disease compared to the AFEP alone. A pilot evaluation of this project demonstrated feasibility as well as improvements in some health behaviors [38]. Participants in the trial (*n* = 462) were located at 49 different community sites and were recruited locally by site personnel and screened for eligibility by study personnel. Forty-six sites were located in Allegheny County, and three sites were located in Washington, Mercer, and Fayette counties, respectively. The sites included eight churches, two YMCA sites, eight subsidized housings, four community centers, 13 senior centers, four fitness clubs or centers, six residential facilities, and four libraries. Participants were eligible for the research study if they were age 50 years or older and did not have medical contraindications including oxygen use, hospitalization for a cardiac event, or major surgery in the previous six months.

For this analysis, we included 242 women who completed the PRESS scale at their baseline visit, between April 2012 and April 2014. The trial also included men but we chose to restrict this study to women as the presence of gender-specific questions in the scale demanded separate evaluations for men and women and we did not have an adequate sample for evaluating men separately. We also excluded participants who did not respond to one or more items on the scale (112 out of 354 women who were administered the PRESS scale). Participants completed the self-efficacy scale as part of a larger battery of self-administered items (including the Western Ontario and McMaster Universities Arthritis Index (WOMAC)) at the intervention site in the community or at home. Written informed consent was obtained from all participants in the study. The study was approved by the University of Pittsburgh Institutional Review Board and the Ethics committee.

For this analysis, we excluded the item assessing self-efficacy for smoking cessation because 92.8 % of the participants did not smoke. We also excluded one item which was specific only to men (i.e., how confident are you that you can get advice on prostate cancer screening?). Our final analysis sample included 242 women and 19 self-efficacy items.

### Statistical analysis

All analyses were performed using SPSS version 20 (SPSS Inc., Chicago, IL) and SAS software, version 9.3 (SAS Institute Inc, Cary, North Carolina). Appropriate descriptive statistics (mean, standard deviation, median, range, frequency or percentage) were employed to summarize participant characteristics and self-efficacy data. Inter-item correlations were calculated using Pearson correlation coefficients to investigate the interrelationships and possible clustering among the items. An exploratory factor analysis (EFA) with principal axis factoring (PAF) extraction and orthogonal varimax rotation (factors assumed to be uncorrelated) was used to explore the underlying structure of the scale. The PAF method searches for the fewest number of factors that can explain the variance in the item set [39], and is preferred over principal component analysis because it provides an extraction of the shared variance among the items (rather than a combination of unique and shared variance) [40]. Although we performed both oblique and orthogonal rotations, our results were very similar. We chose to report the orthogonal results, which are parsimonious, simpler to understand, and more replicable [41]. Kaiser-Meyer-Olkin measure [42] and Bartlett’s test of sphericity [43] were used to evaluate sampling adequacy and the appropriateness of conducting PAF. A Kaiser-Meyer-Olkin value of  ≥0.60 was used to indicate an adequate sample and a significant Bartlett’s test was used to indicate appropriateness of PAF. In the PAF solution, the Cattell’s scree test and the total variance explained were examined to determine the number of underlying factors in the PRESS Scale and factors with eigenvalues greater than 1.0 were extracted. In the extraction phase, items that met a minimum factor loading of .50 were considered relevant [44]. Items with loading ≥ .50 on more than one factor were considered cross-loading items [45]. If the items did not load on any factor at the cut-off of .50, the item was flagged for further investigation.

After the EFA was conducted, a parallel analysis was performed to determine and confirm the appropriate number of factors to retain [46]. A random dataset with the same sample size (*n* = 242) and number of variables (*n* = 19) as the original dataset was first generated with 1000 repetitions. Next, the mean and 95^th^ percentile of each eigenvalue of the random data was calculated. Factors in the PAF results with eigenvalues larger than the mean eigenvalues of the parallel analysis were retained [46].

Subscales were interpreted and labeled by the research team based on the factors identified by this PAF approach. Mixed models were used to take into account the clustered effect of data with patients nested in program sites (i.e., program sites were entered as random effects using SAS PROC MIXED and GLIMIX) and to test differences in base line characteristics and self-efficacy in the use of preventive services between subgroups. Finally, internal consistency of the subscales was assessed by using Cronbach’s alpha coefficient, a reliability index that estimates the internal consistency of the items in the instrument [47]. Alpha coefficients and item-total correlations were examined. An alpha coefficient of .80 or higher was considered to be acceptable [48].

## Results

### Sample characteristics

Among 242 women participants, the mean age was 72 years, 79 % were white and 18 % were black, 65 % had some college or higher education, 86 % had a self-reported diagnosis of arthritis, and 31 % reported exercising regularly before the program started (Table 1).

**Table 1:** Participants characteristics of 242 women participants

Variable^a^	Descriptive statistics
Mean age (SD, range)	72.2 (7.4, 54–90)
Race (%)	
White	188 (79.4)
Black	43 (18.1)
Other	6 (2.5)
Education (%)	
High school or less	83 (34.7)
Some college or higher	156 (65.3)
Diagnosis of arthritis (%)	206 (86.2)
Pre-program exercise routine (%)	
Never exercise	28 (12.1)
Exercise sometimes	131 (56.7)
Exercise regularly	72 (31.0)

^a^Number of missing values in race (*n* = 5), education (*n* = 3), diagnosis of arthritis (*n* = 3), pre-program exercise routine (*n* = 11)

### Item characteristics

The item characteristics of the PRESS Scale are summarized in Table 2. Mean scores of the PRESS scale items varied from 6.4 to 9.6, with standard deviations varying from 1.3 to 3.3.

**Table 2:** Item characteristics of the 19-item preventive services use self-efficacy scale (*N* = 242)

How confident are you that you can…	Mean (SD)	Median (IQR)	% of minimum scores (1–2)	% of maximum score (9–10)
1.do gentle exercises for muscle strength 2–3 times/week?	8.7 (1.9)	10.0 (2)	2.1	70.2
2.do gentle exercises for flexibility?	8.7 (1.8)	10.0 (2)	1.2	67.4
3.do moderate physical activity for at least 2 1/2 h/week?	6.4 (3.3)	7.0 (6)	20.7	35.1
4.exercise without making symptoms worse?	7.5 (2.5)	8.0 (4)	4.1	42.2
5.get information about disease and disability prevention from community resources?	7.5 (2.7)	8.0 (4)	7.4	46.3
6.ask your doctor things about health issues that concern you?	9.3 (1.4)	10.0 (1)	0.4	82.6
7.discuss openly with your doctor any personal problems that may be related to your health?	9.1 (1.6)	10.0 (1)	0.8	77.4
8.work out differences with your doctor when they arise?	9.0 (1.7)	10.0 (1)	1.2	76.5
9.continue to do the things you like to do with friends and family?	8.9 (1.8)	10.0 (2)	0.8	70.6
10.keep from feeling sad or down in the dumps?	8.1 (1.9)	8.5 (3)	1.2	50.0
11.take an active role to manage your systolic blood pressure?	8.9 (1.4)	9.0 (2)	0.4	67.8
12.take an active role to manage your blood glucose (sugar) level?	8.7 (1.6)	9.0 (2)	0.4	65.7
13.take an active role to manage your LDL cholesterol level?	8.5 (1.7)	9.0 (2)	0.8	62.0
14.get a colonoscopy test?	9.2 (2.1)	10.0 (0)	3.7	83.1
15.get an influenza vaccine?	9.4 (1.9)	10.0 (0)	4.1	90.9
16.get a pneumonia vaccine?	9.5 (1.8)	10.0 (0)	2.9	89.7
17.get a Mammogram? (women specific)	9.6 (1.3)	10.0 (0)	1.2	92.2
18.get a Pap test and pelvic exam? (women-specific)	9.5 (1.5)	10.0 (0)	1.7	88.4
19.get a bone density test? (women-specific)	9.4 (1.8)	10.0 (0)	2.9	86.4

### Exploratory factor analysis

Prior to performing the exploratory factor analysis, the suitability of the data for factor analysis was assessed. The inter-item correlations varied from .009 (item 13: take an active role to manage your LDL cholesterol level and item 15: get an influenza vaccination) to .90 (item 1: do gentle exercises for muscle strength and item 2: do gentle exercise for flexibility). The correlation matrix was factorable because all items correlated ≥ .30 with at least three other items in the matrix. In addition, a Kaiser-Meyer-Olkin measure of sampling adequacy of .81 and Bartlett’s test of sphericity (*p* < .001) also suggested that the initial extraction process could be continued. The PAF analysis of the PRESS Scale among 242 subjects who answered all the questions suggested five underlying factors with eigenvalues from 1.10 to 6.0.

**Fig. 1:**
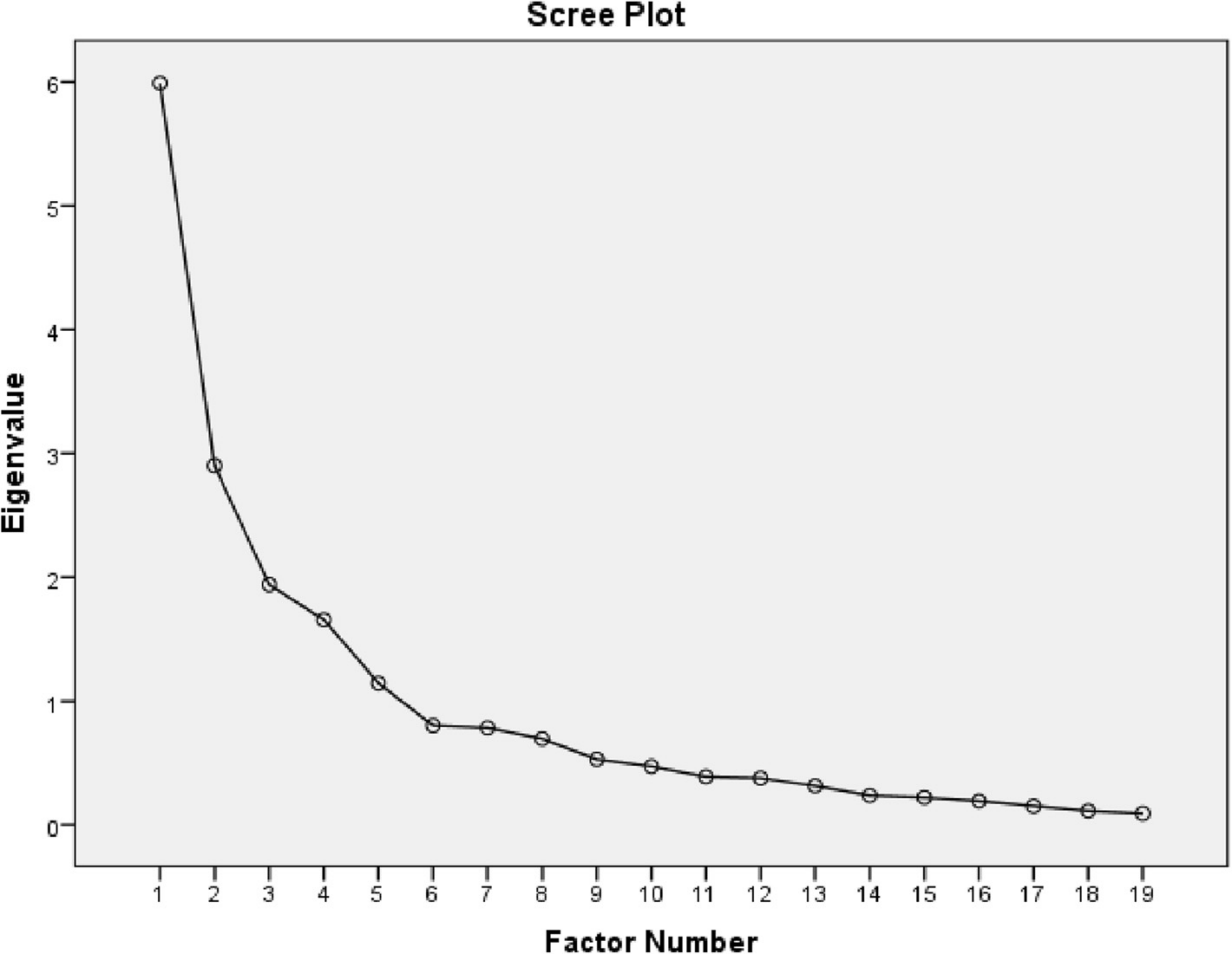
Scree plot of the exploratory factor analysis of the PRESS scale

Table 3 shows the PAF results with orthogonal varimax rotation. There were no cross-loadings, and the factor loadings varied between .52 and .92. The Scree plot (Fig. 1) indicated a 5 factor solution. Five factors were interpreted and labeled “Factor I: Self-efficacy for Exercise Subscale (items 1, 2, 3 and 4)”, “Factor II: Self-efficacy for Communication with the Physician Subscale (items 6, 7, and 8)”, “Factor III: Self-efficacy for Self-Management of Chronic Disease Subscale (items 11, 12, and 13)”, “Factor IV: Self-efficacy for Obtaining Screening Tests Subscale (item 14, 17, 18 and 19)” and “Factor V: Self-efficacy for Getting Vaccinations Regularly Subscale (items 15 and 16)”. These factors explained 64 % of the total variance in the rotated PAF results. The item communalities, which provide information on how much item variance is explained by the extracted factors, ranged in value between .22 (item 5) to .87 (item 7). Three items (item 5, 9, and 10) did not have loadings at the .50 value on any factors (highest factor loading ranged from .32 to .47). These items also had the lowest communalities (.22, .35, .51, respectively), and were flagged for further investigation and discussion.

**Table 3:** Factor loadings and total variance explained from the factor structure matrix (Varimax rotation) for the 16 Items in the preventive services use self-efficacy scale

	Factor				
Item by factor	I	II	III	IV	V
How confident are you that you can… Factor I. Self-efficacy for Exercise					
1.do gentle exercises for muscle strength 2–3 times/week?	.87				
2.do gentle exercises for flexibility?	.83				
3.do moderate physical activity for at least 2 1/2 h/week?	.63				
4.exercise without making symptoms worse?	.67				
Factor II. Self-efficacy for Communication with Physicians					
6.ask your doctor things about health issues that concern you?		.86			
7.discuss openly with your doctor any personal problems that may be related to your health?		.92			
8.work out differences with your doctor when they arise?		.88			
Factor III. Self-efficacy for Self-Management of Chronic Disease					
11.take an active role to manage your systolic blood pressure?			.76		
12.take an active role to manage your blood glucose (sugar) level?			.86		
13.take an active role to manage your LDL cholesterol level?			.81		
Factor IV. Self-efficacy for Obtaining Screening Tests					
14.get a colonoscopy				.55	
17.get a mammogram				.70	
18.get a Pap test and pelvic exam				.85	
19.get a bone density test				.52	
Factor V. Self-efficacy for Getting Vaccinations Regularly					
15.get an influenza vaccine?					.72
16.get a pneumonia vaccine?					.89
% of Variance	15.5	14.8	12.9	10.5	9.8
Cumulative %	15.5	30.3	43.2	53.7	63.5

Note: Three items did not load on any of 5 factors: item 5 (get information about disease and disability prevention from community resources?), item 9 (continue to do the things you like to do with friends and family?), and item 10 (keep from feeling sad or down in the dumps?)

To ensure the robustness of these factors, the items (items 5, 9, and 10) with lowest communalities were removed from the final factor pattern matrix and yielded similar results (data not shown). Similar findings were observed from the oblique promax rotation (data not shown). The parallel analysis also supported a five-factor solution.

### Self-efficacy and participant characteristics

As shown in Table 4, women with arthritis had a lower score in the “Self-efficacy for Exercise Subscale” than those without arthritis (30.8 ± 7.9 vs. 36.4 ± 4.2, *p* = <.001). Older women who exercised regularly compared to those who did not exercise regularly had higher scores in the “Self-efficacy for Exercise Subscale” (33.9 ± 6.6 vs. 26.9 ± 9.6, *p* = .004). Older women with self-reported hypertension (25.7 ± 4.6 vs. 27.1 ± 3.8, *p* = .02) or hypercholesterolemia (25.5 ± 4.6 vs. 27.1 ± 4.0, *p* = .006) had lower scores in the “Self-efficacy for Self-Management of Chronic Disease Subscale” than those without these chronic disorders. Women who received flu shots regularly (19.7 ± 1.3 vs. 16.2 ± 6.0, *p* < .0001) or a pneumonia shot (19.4 ± 2.1 vs. 16.7 ± 5.8, *p* < .0001) had higher scores in the “Self-efficacy for Getting Vaccinations Regularly Subscale” than those who did not. The stratification analyses found no difference in the five subscales by race, and education (data not shown).

**Table 4:** Preventive services use self-efficacy subscales among different subgroups

Factors or self-efficacy subscales (Number of items^a^)	N	Mean (SD)	Median (Range, IQR)
*Self-efficacy for Exercise (4)*			
By diagnosis of arthritis			
Yes	206	30.8 (7.9)****	32.0 (6–40, 10)
No	27	36.4 (4.2)	37.0 (24–40, 6)
By pre-program exercise routine			
Never exercise	28	26.9 (9.6)***	29.0 (6–40, 14.5)
Exercise sometimes	131	30.9 (7.4)	32.0 (8–40, 11)
Exercise regularly	72	33.9 (6.6)	36.0 (11–40, 9.5)
*Self-efficacy for Self-management of Chronic Disease (3)*			
By self-reported hypertension			
Yes	165	25.7 (4.6)*	27.0 (3–30, 7)
No	77	27.1 (3.8)	28.0 (12–30, 4)
By self-reported diabetes			
Yes	56	25.8 (4.5)	27.5 (14–30, 7)
No	186	26.2 (4.4)	27.0 (3–30, 6)
By self-reported hypercholesterolemia			
Yes	136	25.5 (4.6)**	26.5 (3–30, 7)
No	99	27.1 (4.0)	29.0 (12–30, 5)
By numbers of comorbidities			
>3	111	26.0 (4.1)	27.0 (14–30, 7)
≤3	131	26.2 (4.6)	27.0 (3–30, 6)
Factors or Self-Efficacy Subscales (Number of items^a^)	N	Mean (SD)	Median (Range, IQR)
Self-efficacy for *Getting Vaccinations Regularly (2)*			
By having a flu shot in the last year at baseline			
Got a flu shot in the last year	189	19.7 (1.3)****	20.0 (7–20, 0)
Did not get a flu shot in last year	51	16.2 (6.0)	20.0 (2–20, 7)
By having a pneumonia shot at baseline			
Had a pneumonia shot previously	185	19.4 (2.1)****	20.0 (2–20, 0)
Never had a pneumonia shot	46	16.7 (5.8)	20.0 (2–20, 4)

^a^The score for each item ranges 1 to 10

*:*P* < .05, **:*P* < .01, ***:*P* < .001, ****:*P* < .0001

### Internal consistency and correlations between subscales

Table 5 presents the descriptive statistics between factor correlations and Cronbach’s alpha coefficients for the five generated subscales (corresponding to Factors I to V). The internal consistency ranged from .81 to .94. All PRESS subscales demonstrated good internal consistency. The correlations among the subscales ranged from .11 to .53 (Table 5). The item-total correlations were high for all items within the subscales: “Self-efficacy for Exercise (.81 to .84)”, “Self-efficacy for Communication with Physicians (.93 to .95)”, “Self-efficacy for Self-management of Chronic Disease (.88 to .93)”, “Self-efficacy for Obtaining Screening Tests (.73 to .83)”, and “Self-efficacy for Getting Vaccinations Regularly (.91 to .93)”.

**Table 5:** Internal consistency and factor correlations of the preventive services use self-efficacy subscales (*N* = 242)^a^

Factors or self-efficacy subscales (Numbers of items)	Cronbach’s alpha	Mean (SD)	Median (Range, IQR)	Factor			
				I	II	III	IV
Factor I: Self-efficacy for Exercise (4)	.86	31.4 (7.9)	33.0 (4–40, 11)				
Factor II: Self-efficacy for Communication with Physicians (3)	.94	27.5 (4.4)	30.0 (4–30, 3)	.28			
Factor III: Self-efficacy for Self -management of Chronic Disease (3)	.89	26.1 (4.4)	27.0 (3–30, 6)	.37	.34		
Factor IV: Self-efficacy for Obtaining Screening Tests (4)	.81	37.7 (5.3)	40.0 (4–40, 2)	.15	.21	.28	
Factor V: Self-efficacy for Getting Vaccinations Regularly (2)	.83	18.9 (3.3)	20.0 (2–20, 0)	.11	.20	.11	.53

*IQR* Interquartile range

^a^Higher number indicates higher self-efficacy (range: 1–10 for each item)

## Discussion

We identified a five-factor structure with good internal consistency in the PRESS scale among older women participating in a community-based cluster randomized trial. These five factors were “Self-efficacy for Exercise”,”Self-efficacy for Communication with Physicians”, “Self-efficacy for Self-management of Chronic Disease”, “Self-efficacy for Obtaining Screening Tests”, and “Self-efficacy for Getting Vaccinations Regularly”. Self -administration of this scale was found to be acceptable and feasible among older women in community settings.

From the original 19 items that underwent factor analysis, we identified a five-factor structure consisting of 16 items. The three items that did not load adequately dealt with self-efficacy in preventing depression, maintaining social contact, and obtaining information regarding disease and disability prevention. These items, which had been added to the scale to represent multiple methods of prevention, performed poorly as elements of this instrument and results indicated that these items need to be removed from the PRESS scale. This decision does not indicate that these items represent less important prevention methods, but that they are not statistically relevant to form a factor or subscale in this model of self-efficacy for the use of preventive services. Preventing depression and maintaining social contact may not be perceived as a ‘preventive service or behavior’ that needs to be actively accessed (as opposed to exercise which is often perceived as one) and could be the reason why it did not become part of the structure of the final scale. Similarly, obtaining information regarding disease and disability prevention from community resources seems to be part of a self-efficacy construct distinct from that of routine preventive services use by older women.

Five subscales in the final PRESS scale showed good internal consistency, indicating the reliability of this scale. The high item-total correlations indicate that the score on each item is consistent with the averaged score for the other questions, implying that all the items are integral to the scale and represent the same construct. Subgroups with a particular chronic disease (hypertension, hypercholesterolemia, and arthritis) had lower self-efficacy subscale scores for accessing preventive services relevant to the disease and those who were accessing a preventive service (flu shot, pneumonia shot) had higher self-efficacy scores in that subscale, providing further support for the construct validity of the 16-item PRESS scale.

Our study has some limitations. Firstly, our sample consisted mostly of older white women, which may limit generalizability of our results to other populations (e.g., African Americans). Secondly, the self-efficacy questions were asked alongside questions on preventive behaviors, but this is likely to have resulted in more accurate self-efficacy reporting than a reporting bias. In future, we propose to do confirmatory studies with more diverse samples to verify our results and explore the need for additional items to strengthen the scale. Other potential future analyses may focus on the ability of the PRESS scale to identify high-risk individuals in need of improving self-efficacy in preventive behaviors and the use of preventive services.

To our knowledge, the PRESS scale is the first instrument developed to measure self-efficacy in a wide range of preventive behaviors and preventive services use among older women. Older adults are a high risk for disease and disability and it is important to improve access to, and utilization of, all proven prevention methods that support healthy aging and a good quality of life. Scales that measure self-efficacy in the management of specific chronic diseases do not capture this self-efficacy domain comprehensively. Given that older adults are likely to have multiple chronic conditions and impairments, this multi-pronged measure would provide consolidated information on the broad set of prevention strategies relevant to the health of older adults. The five self-efficacy subscales identified in the EFA represent crucial factors in the prevention of morbidity and mortality. Compared to the Stanford scale which evaluates self-efficacy in the management of chronic diseases, the PRESS scale is more comprehensive and specific for evaluating self-efficacy in the use of preventive services among older women. Given the current evidence and ongoing policy emphasis, the PRESS scale may serve as an important tool to identify older women with low self-efficacy in the use of preventive services and to evaluate related interventions.

## Conclusions

The 16-item PRESS scale demonstrated preliminary evidence of being a valid and reliable tool to measure self-efficacy in the use of preventive services among older women. It is acceptable and feasible for administration in community settings and can potentially be used to evaluate the impact of interventions designed to improve self-efficacy in the use of preventive services in community-dwelling older women.

## Abbreviations

PRESS: Preventive services use self-efficacy
CAPH-PRC: Center for aging and population health - prevention research center
LDL-C: Low-density lipoprotein cholesterol
AFEP: Arthritis foundation exercise program
EFA: Exploratory factor analysis
PAF: Principal axis factoring
